# Sleep structure and autonomic nervous system state during four months of isolation in a space analogue mission

**DOI:** 10.3389/fnhum.2026.1720237

**Published:** 2026-04-14

**Authors:** Martin Glos, Matthew Salanitro, Naima Laharnar, Artem Demin, Thomas Penzel, Ingo Fietze

**Affiliations:** 1 Interdisciplinary Center of Sleep Medicine, Charité – Universitätsmedizin Berlin, Berlin, Germany; 2 Russian Federation State Research Center, Institute of Biomedical Problems, Russian Academy of Science, Moscow, Russia; 3 Department of Medicine, Southwest Medical University Affiliated Zigong Hospital, Zigong, Sichuan, China

**Keywords:** astronauts, autonomic nervous system, biomarkers, heart rate variability, sleep, space-analogue mission, sleep fragmentation

## Abstract

**Introduction:**

Insufficient or disturbed sleep impairs nocturnal physiological recovery and may negatively affect autonomic nervous system (ANS) regulation. Astronauts are a particularly vulnerable group due to sustained workloads, isolation, and exposure to extreme operational conditions. The present study investigated the interaction between sleep structure and ANS state under different sleep conditions during a prolonged space analogue mission (SAM) conducted as part of the *Scientific International Research in a Unique Terrestrial Station* (SIRIUS-19) project.

**Methods:**

Six healthy participants (three men and three women; age: 34.3 ± 5.7 years) were studied over eight nights during the four-month SIRIUS-19 mission: one night of undisturbed sleep pre-isolation, one night of undisturbed sleep post-isolation, and six nights during the isolation phase comprising three undisturbed nights, one night of complete sleep deprivation, and two nights of experimentally induced sleep fragmentation (repeated short awakenings vs. one prolonged awakening). Sleep structure and ANS state were assessed using a portable, self-applicable, medical-grade sleep recording system that captured electroencephalography (EEG), electrooculography (EOG), electrocardiogram (ECG), and plethysmography signals.

**Results:**

Significant differences in total sleep time were observed across different nights (*p* = 0.003). On nights of undisturbed isolation, participants achieved more than 7 h of sleep, while nights with sleep fragmentation was associated with reduced sleep efficiency (<80%). ANS state parameters differed significantly across conditions, including the pulse rate (PR) (*p* < 0.0001) and the heart rate variability (HRV) LF/HF ratio (*p* < 0.005), with the most pronounced autonomic activation occurring during the night of complete sleep deprivation.

**Discussion:**

Using a portable monitoring approach, this study demonstrates that nocturnal ANS regulation during prolonged isolation is relatively resilient to moderate sleep fragmentation, but is markedly affected by sustained sleep loss. These findings highlight the importance of preserving restorative sleep continuity when planning operationally demanding space missions and support the feasibility of portable sleep and ANS monitoring in extreme environments.

## Introduction

The autonomic nervous system (ANS) is a central regulatory system responsible for the continuous control of cardiovascular function and the maintenance of physiological stability in humans (Gordan et al., 2015). By integrating signals from the central nervous system and peripheral organs, the ANS continuously adjusts the heart rate, vascular tone, and blood pressure in response to physical, cognitive, and emotional demands. This regulation allows individuals to respond effectively to stressors while maintaining internal balance under both resting and challenging conditions (Dampney, 2016). The sympathetic branch of the ANS primarily supports arousal and energy mobilization during periods of increased demand, whereas the parasympathetic branch promotes recovery, restoration, and energy conservation (Gordan et al., 2015). The balance between these two branches is therefore essential for cardiovascular stability, stress resilience, and sustained functional capacity.

When this balance is disrupted, it can lead to a persistent state of strain on the ANS, typically characterized by an overactive sympathetic response and reduced parasympathetic modulation (Borovac et al., 2020). Such an ANS state imbalance has been associated with impaired cardiovascular regulation, increased vulnerability to stress, and a higher risk of adverse health outcomes, including elevated morbidity and all-cause mortality rates (Oliver et al., 2020). Importantly, alterations in ANS activity may occur even in otherwise healthy individuals when exposed to prolonged or repeated stressors, making ANS state regulation a sensitive indicator of physiological strain in demanding environments (Jarczok et al., 2013).

Sleep plays a fundamental role in the restoration and stabilization of ANS function and is essential for maintaining cardiovascular regulation (Dai et al., 2024). During healthy, consolidated sleep, ANS state shifts toward parasympathetic predominance, supporting cardiovascular recovery and reducing physiological stress accumulated during wakefulness. Both sufficient sleep duration and good sleep continuity are therefore critical for effective ANS recovery (Fink et al., 2018). In contrast, insufficient sleep, sleep restriction, and fragmented sleep have been shown to impair the nocturnal regulation of ANS state, leading to increased sympathetic activity and reduced parasympathetic modulation (Meerlo et al., 2008). Such alterations reflect a lack of complete physiological recovery, which may extend into daytime functioning.

Evidence from experimental and epidemiological studies indicates that disturbed or non-restorative sleep is associated with impaired ANS regulation and increased cardiovascular risk, even in otherwise healthy individuals (Tobaldini et al., 2017). Although the effects of acute and chronic sleep restriction on ANS state have been relatively well studied, considerably less is known about the specific impact of sleep fragmentation on nocturnal ANS state (Messa et al., 2024). Notably, the combination of reduced sleep duration and repeated sleep interruptions appears to pose a particularly high risk for autonomic imbalance, highlighting the importance of sleep continuity for maintaining a stable ANS state (Tobaldini et al., 2017).

In occupations characterized by high cognitive load, sustained vigilance, and limited opportunities for recovery, disturbances in sleep and ANS regulation may result in particularly serious consequences (Jarczok et al., 2013). Professions such as emergency services and military place continuous demands on physiological and psychological resilience, making stable sleep–wake regulation and ANS state balance critical for performance and safety. In these settings, impaired sleep and incomplete autonomic recovery can lead to cumulative fatigue, reduced cognitive efficiency, and increased vulnerability to stress, potentially compromising decision-making and operational effectiveness (Jarczok et al., 2013; Matsangas and Shattuck, 2020; Stout et al., 2021).

Importantly, spaceflight and space analogue missions (SAMs) can be viewed as an extension of these terrestrial high-demand environments. They incorporate many of the same stressors but expose individuals to these challenges continuously and over prolonged periods, often under conditions of isolation and confinement. Astronauts represent a particularly vulnerable occupational group, as space missions combine extreme environmental conditions with prolonged periods of isolation and confinement (Jones et al., 2022; Kuldavletova et al., 2023; Sy et al., 2023). Factors such as high workload, altered light–dark exposure, restricted living space, and psychosocial stress can disrupt circadian rhythms and sleep–wake regulation, thereby challenging ANS state stability (Flynn-Evans et al., 2025; Tomaraei et al., 2025). Evidence from studies on spaceflight and space analogue missions indicates that astronauts frequently experience reduced sleep duration, fragmented sleep, and impaired sleep quality, particularly during the adaptation phases of their missions (Monk et al., 1998; Gundel et al., 2001; Basner et al., 2014). During long-duration missions or isolation experiments, these disturbances may accumulate over time, increasing the risk of sustained ANS state imbalance and insufficient physiological recovery.

The present study is part of the space research program *Scientific International Research in a Unique Terrestrial Station* (SIRIUS-19), implemented by the IMBP RAS and NASA HRP. This program involves a four-month SAM (Belakovsky et al., 2019; NASA, 2026). Within this highly controlled, yet operationally realistic setting, prolonged isolation, confinement, and mission-like workloads were used to simulate key aspects of long-duration spaceflight, excluding the effects of microgravity. Building on previous short-term laboratory and SAM studies, this investigation extends existing research by examining sleep and ANS state under long-term isolation conditions that more closely resemble the temporal and environmental demands of real space missions (Laharnar et al., 2020; Schlagintweit et al., 2023). In particular, the study combined repeated nocturnal assessments of sleep structure with continuous measures of ANS state across different sleep conditions, including undisturbed sleep, sleep fragmentation, and complete sleep deprivation, all observed within the same individuals over the course of the mission.

A further novel aspect of this study is the use of a portable, self-applicable, medical-grade recording system to assess sleep and ANS state parameters in a remote, confined environment over several months. This approach enables the investigation of physiological regulation under conditions where conventional laboratory-based measurements are not feasible. It also provides important insights into the applicability and reliability of such systems for future space missions and other extreme operational settings.

The primary aim of this study was to investigate the interplay between sleep and ANS state under experimentally disturbed and undisturbed sleep conditions during a prolonged SAM. Specifically, we sought to determine how different types of sleep disruption, such as sleep fragmentation and complete sleep deprivation, affect nocturnal ANS state and whether these effects differ in their magnitude and pattern. A secondary aim was to evaluate the feasibility and robustness of using a portable, self-applicable recording system to assess sleep and ANS state under long-term isolation conditions.

## Methods

### Measurement environment and study participants

The study was conducted as part of the SIRIUS-19 project at the medical-technical Ground Test Complex (NEK), a ground-based isolation facility designed to simulate long-duration spaceflight conditions. The facility comprises interconnected pressurized modules providing a confined and controlled environment in which crew members are exposed to prolonged isolation, restricted living space, and mission-like operational demands.

Environmental and operational conditions within the facility were standardized throughout the mission to replicate key aspects of interplanetary flight scenarios. Work–rest schedules, communication conditions, and daily activities were strictly regulated. Artificial lighting was used to maintain a structured day–night cycle, with scheduled light exposure of at least 400 lux from 07:00 to 23:00, aligned with predefined sleep–wake periods. Individual crew quarters were equipped with personal lighting controls, allowing participants to switch lights on or off as needed. Diet and physical activity followed the mission schedule, with standardized meals aligned with a regular 24-h sleep–wake cycle and countermeasure exercises to compensate for reduced physical activity during confinement (Koschate et al., 2022). To minimize acute effects on sleep and ANS measurements, no strenuous physical activity or unusual dietary intake occurred in the evening prior to sleep recording nights. Further technical details of the infrastructure and mission timeline have been described previously (Saveko et al., 2021; Supolkina et al., 2021; Pauly et al., 2025).

A total of six crew members participated in the study, including one crew commander who had previously been to the International Space Station (ISS), one flight engineer, one crew physician, and three researchers who had already participated in SAM/isolation experiments. The crew consisted of three male and three female individuals (*m* = 3/*f* = 3), with four Russian and two American participants, aged between 28 and 44 years (mean age 34.3 ± 5.7 years). The mean body mass index (BMI) was 22.5 ± 2.8 kg/m^2^, and the Apnea–Hypopnea Index (AHI) averaged 2.4 ± 1.2 events/h. Prior to inclusion, all participants underwent comprehensive medical screening to exclude cardiovascular disease, sleep disorders, and other relevant health conditions. None of the participants was taking regular medication at the time of the study. All participants were recruited in a healthy state and reported no sleep complaints (Belakovsky et al., 2019; NASA, 2026). The study protocol was reviewed and approved by the Biomedicine Ethics Committee of the RF SRC—Institute of Biomedical Problems, Russian Academy of Sciences; the Physiological Section of the Russian Bioethics Committee; and the Russian Federation National Commission for UNESCO (Protocol number: #501; February 18, 2019). Each participant provided written informed consent before participating.

### Materials

Sleep structure and nocturnal cardiovascular signals were recorded using the portable, medical-grade sleep recorder SOMNOtouch RESP (Somnomedics AG, Randersacker, Germany). Self-applicable electrodes positioned at EEG-Fp1, EEG-M2, EOG-left, and EOG-right were used to assess sleep patterns. Electrocardiogram (ECG; single lead) and plethysmography for SpO₂ (finger clip) were recorded for the subsequent analysis of ANS state.

From the recordings, sleep-related variables—including sleep onset latency (SOL), total sleep time (TST), sleep efficiency (SE), and the relative proportion of sleep stages—were derived. In addition, cardiovascular and autonomic parameters were derived as mean values across the night, including beat-to-beat RR intervals (RR), heart rate variability (HRV), the low-to-high frequency spectral power ratio of HRV (HRV LF/HF), pulse rate (PR), pulse transit time (PTT), systolic blood pressure (SBP), and diastolic blood pressure (DBP).

In addition to physiological recordings, subjective sleepiness was assessed using the Karolinska Sleepiness Scale (KSS), a 9-point self-rating scale ranging from 1 (extremely awake) to 9 (very sleepy, fighting sleep) (Akerstedt et al., 2014).

### Protocol

This study employed a within-subject, repeated measures design to assess the effects of different sleep conditions on ANS regulation during a prolonged SAM. The study was planned, approved, and funded as a collaborative research project between the German Space Agency (DLR) and the State Scientific Center of the Russian Federation (SSC RF), with grant no. DLR 50WB1735. From the outset, it was an integral part of the SIRIUS-19 experiment (Belakovsky et al., 2019).

The protocol was designed to enable reliable assessment of different sleep conditions under controlled isolation. It enabled assessment of undisturbed sleep (Normal), complete sleep deprivation (No sleep), and sleep fragmentation, with fragmentation implemented in two forms: Fragmentation 1 (two brief awakenings) and Fragmentation 2 (one prolonged awakening). To minimize carryover effects, a minimum washout period of 1 week was ensured following nights with impaired sleep. In addition, sleep recording nights were scheduled so that they did not follow other experimental procedures that could interfere with nocturnal sleep or physiological recordings. All six participants were assessed in parallel across eight nights distributed over the four-month isolation period (Table 1).

**Table 1:** Timeline of investigations.

Night	Sleep condition	Day of mission	Description
Night 1	Normal, pre-isolation	- 5	Pre- isolation sleep: Undisturbed sleep in the facility with doors kept open.
Night 2	Normal, isolation	5	Undisturbed sleep.
Night 3	No sleep, isolation	54	Forced complete sleep deprivation: At midnight, all crew members were required to unload a cargo ship.
Night 4	Normal, isolation	71	Undisturbed sleep.
Night 5	Fragmentation 1, isolation	91	Two brief awakenings (02:00 h and 04:00 h), during which the lights were turned on and a short radio broadcast announced that the supply ship would be arriving shortly.
Night 6	Fragmentation 2, isolation	108	One long awakening lasting 1 h (03:15–04:15 h), during which the lights were turned on and a constant, disturbing radio signal was present.
Night 7	Normal, isolation	117	Undisturbed sleep.
Night 8	Normal, post-isolation	120 + 5	Post-isolation sleep: Undisturbed sleep in the facility with doors kept open.

For each study night, crew members self-applied the electrodes and sensors on site prior to going to bed. The KSS was completed in the evening before sleep onset and again upon awakening in the morning. Sleep-related recordings were obtained using the same experimental setup throughout the study period to ensure consistency across conditions.

Participants were assessed under normal sleep conditions 1 week before and 1 week after the isolation period. These baseline and post-isolation measurements were conducted in the same rooms used during isolation, but without confinement (doors open), to allow comparison with isolation conditions while minimizing environmental differences.

During the isolation phase, nocturnal measurements were conducted on six occasions under different sleep conditions: Three nights under normal sleep conditions, two nights with experimentally induced sleep fragmentation, and one night with complete sleep deprivation. Sleep fragmentation was implemented using two distinct protocols. In the first fragmentation condition (Fragmentation 1), participants were awakened twice, at 02:00 and 04:00 h. Awakenings were initiated by switching on the lights and issuing a brief radio announcement indicating that a supply ship was approaching and required unloading, followed shortly by a second announcement canceling the task and switching the lights off. In the second fragmentation condition (Fragmentation 2), participants were awakened once for a continuous period of 1 h starting at 03:15 h, during which the lights were turned on and an aversive acoustic signal was presented. In both fragmentation conditions, participants were allowed to resume sleep following the awakening periods.

The night of complete sleep deprivation simulated an operational scenario in which all crew members were required to unload a cargo ship and therefore remained awake throughout the night. An overview of all recorded nights and experimental conditions is provided in Table 1.

Primary outcomes included the HRV LF/HF ratio and pulse rate as indicators of ANS regulation. Secondary outcomes comprised sleep architecture parameters (e.g., TST, SE, and sleep stages) and subjective sleepiness assessed using the KSS.

### Data analysis

Sleep recordings were processed using the proprietary analysis software Domino (Somnomedics AG, Randersacker, Germany) according to the guidelines of the American Academy of Sleep Medicine (Berry et al., 2018). Sleep-related variables were scored by an experienced sleep technologist. Nocturnal cardiovascular and ANS state parameters were extracted from the same recordings. All statistical analyses were performed using IBM SPSS Statistics. The level of statistical significance was set at *α* = 0.05.

Subjective sleepiness was assessed using the KSS. Evening and morning KSS values were compared within each study night using non-parametric Wilcoxon signed-rank tests, as the small sample size did not allow for reliable assumptions of normality.

Sleep structure and ANS state parameters were analyzed using repeated measures analysis of variance (ANOVA), with night as the within-subject factor representing the eight measurement nights. Assumptions for the repeated-measures ANOVA were evaluated, including interval-scaled data and sphericity assessed using Mauchly’s test. When the assumption of sphericity was violated, Greenhouse–Geisser corrections were applied. Significant main effects were followed by within-subject comparisons between specific experimental conditions. In addition, descriptive analyses were used to characterize changes in sleep architecture and ANS parameters under specific conditions, such as sleep fragmentation and total sleep deprivation.

For continuously recorded ANS parameters, additional time series analyses were conducted. Parameter trajectories were generated in 30-s epochs for each participant and night separately to enable comparisons across sleep-related time segments and experimental conditions.

## Results

### Subjective sleepiness

**Figure 1:**
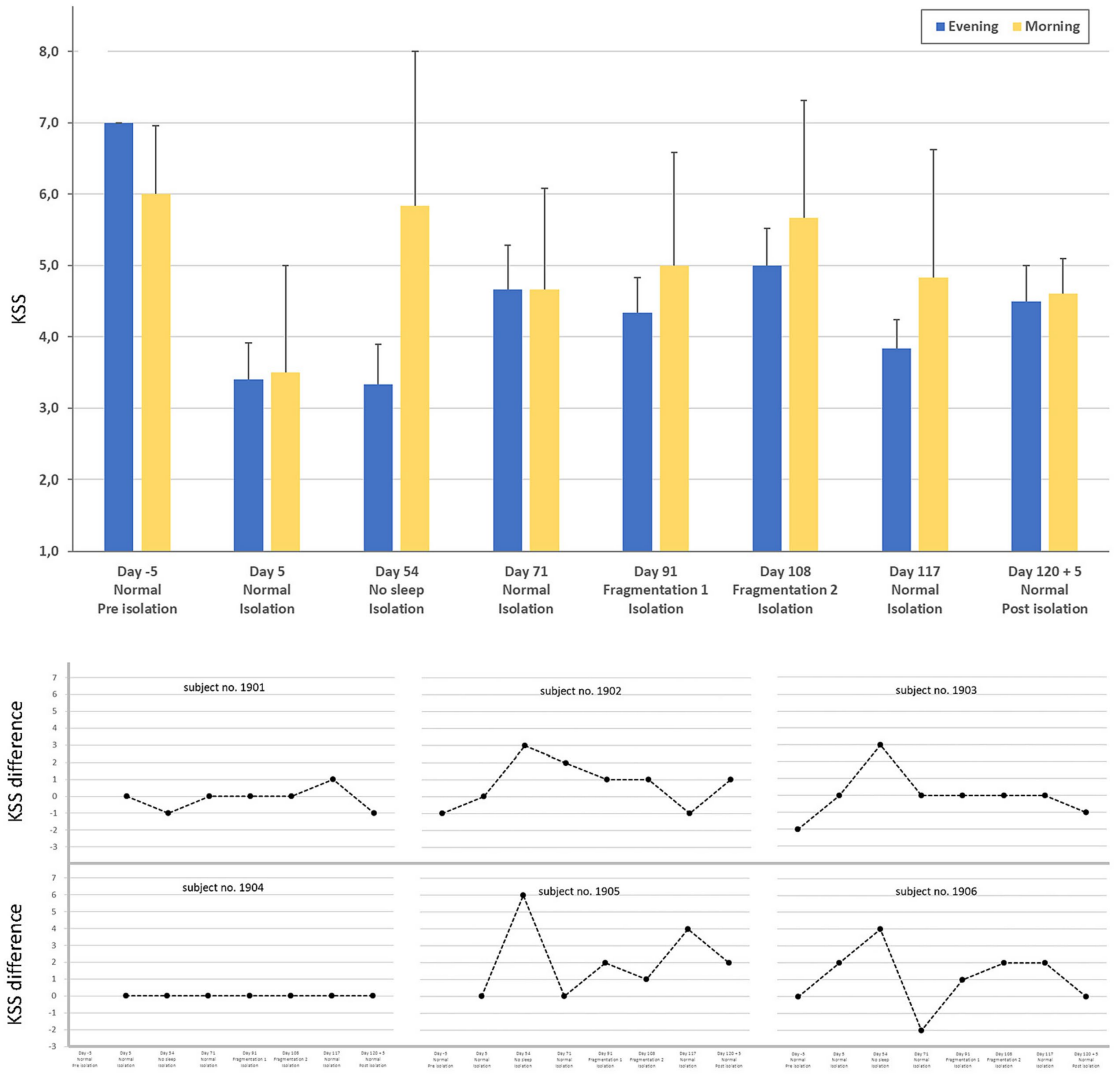
Mean ± SD Karolinska Sleepiness Scale (KSS) ratings for all eight nights, measured before going to bed (Evening) and after waking up (Morning) (upper panel), and the corresponding individual KSS differences between morning and evening (lower panel).

Subjective sleepiness was assessed by comparing evening and morning KSS scores for each study night using Wilcoxon signed-rank tests. Across the study period, no significant differences between evening and morning KSS ratings were observed for any of the nights (Figure 1, upper panel).

However, during the night without sleep (Day 54), participants tended to report higher levels of sleepiness in the morning compared to the preceding evening. Although this difference did not reach statistical significance, a clear trend was observed (*p* = 0.078), with morning KSS scores exceeding evening scores by a mean of 2.5 points. This pattern was evident in four of the six participants (Figure 1, lower panel).

### Sleep structure

With the exception of the night with complete sleep deprivation (Day 54), Table 2 summarizes sleep parameters for each study night and the corresponding within-subject differences. Repeated-measures ANOVA revealed significant differences between nights for TST (*p* = 0.003), indicating marked variability in sleep duration across experimental conditions.

**Table 2:** Sleep parameters for study nights, excluding the night of complete sleep deprivation.

Parameter	Day −5 Normal, pre-isolation	Day 5 Normal, isolation	Day 71 Normal, isolation	Day 91 Fragmentation 1, isolation	Day 108 Fragmentation 2, isolation	Day 117 Normal, isolation	Day 120 + 5 Normal, post-isolation	*p*-value
TIB [min]	376.7 ± 28.8	458.3 ± 14.4	467.5 ± 27.5	446.7 ± 41.4	515.0 ± 35.1	447.5 ± 79.1	442.8 ± 42.3	**0.026**
TST [min]	329.4 ± 30.2	421.0 ± 25.9	430.9 ± 36.4	342.3 ± 42.0	394.9 ± 33.0	360.2 ± 42.7	368.8 ± 55.6	**0.003**
SOL [min]	23.2 ± 21.5	23.6 ± 20.9	12.4 ± 10.5	17.4 ± 21.8	16.4 ± 24.3	51.6 ± 51.2	46.8 ± 61.7	0.332
SE [%]	87.6 ± 7.6	91.9 ± 5.4	92.2 ± 5.7	77.4 ± 13.1	76.8 ± 6.0	82.1 ± 12.9	84.1 ± 15.3	0.125
NREM-N1 + N2 sleep [%-TST]	58.1 ± 14.1	63.9 ± 9.2	66.0 ± 5.3	60.1 ± 3.5	61.5 ± 6.1	59.0 ± 5.1	61.9 ± 7.1	0.415
NREM-N3 sleep [%-TST]	26.0 ± 9.0	15.9 ± 8.6	14.7 ± 3.3	20.3 ± 4.1	18.1 ± 2.2	20.5 ± 6.0	20.2 ± 5.8	0.083
REM sleep [%-TST]	15.9 ± 7.2	20.2 ± 4.6	19.3 ± 3.7	18.9 ± 3.1	20.5 ± 5.3	20.6 ± 2.1	17.9 ± 4.4	0.381
Wake [%-TIB]	20.2 ± 22.6	8.1 ± 5.4	7.8 ± 5.7	34.7 ± 17.0	23.2 ± 6.0	17.9 ± 12.9	17.6 ± 15.7	0.078

TIB, Time in Bed; TST, Total Sleep Time; SOL, Sleep Onset Latency; SE, Sleep Efficiency; min, minutes; %-TST, percentage related to TST; %-TIB, percentage related to TIB; NREM-N1 + N2, sum of Non Rapid-Eye Movement (NREM) sleep stages N1 and N2 amount; NREM-N3 Sleep, NREM sleep stage N3 amount; REM, Rapid-Eye Movement (REM) sleep stage amount. Wake, wake state amount. Mean values +/− standard deviations are shown. Night no. 3 (day 54), without sleep, is not mentioned in the table due to forced complete sleep deprivation. *p*-values for significant differences are plotted in bold.

In particular, mean TST exceeded 7 h on Days 5 and 71, which corresponded to isolation conditions without sleep interruption. In contrast, both nights characterized by sleep fragmentation (Days 91 and 108) were associated with reduced sleep continuity. Descriptive analyses showed that SE declined to values below 80% during these nights.

Sleep stage distribution did not differ significantly across nights. Nevertheless, descriptive inspection of the data revealed that the highest proportions of wakefulness occurred during the two sleep fragmentation nights, suggesting increased sleep disruption under these conditions.

### ANS state parameters

The analysis of ANS state parameters across the eight recording nights revealed significant within-subject differences for PR (*p* = 0.0001), RR (*p* = 0.004), and HRV LF/HF (*p* = 0.005) (Table 3), indicating substantial modulation of autonomic activity across experimental conditions.

**Table 3:** ANS state parameters for all study nights.

Parameter	Day −5 normal, pre-isolation	Day 5 normal, isolation	Day 54 no sleep, isolation	Day 71 normal, isolation	Day 91 fragmentation 1, isolation	Day 108 fragmentation 2, isolation	Day 117 normal, isolation	Day 120 + 5 normal, post-isolation	*P-*value
PR [bpm]	61.7 ± 8.8	57.5 ± 9.3	66.4 ± 6.7	55.8 ± 8.4	54.0 ± 6.6	52.7 ± 6.9	56.3 ± 7.4	61.2 ± 12.1	**0.0001**
HRV-LF/HF [n. u.]	1.6 ± 0.4	1.3 ± 0.4	2.3 ± 0.7	1.5 ± 0.5	1.5 ± 0.5	1.4 ± 0.5	1.5 ± 0.4	1.8 ± 0.4	**0.005**
SBP [mmHg]	118.0 ± 6.8	116.3 ± 14.1	115.8 ± 8.0	112.5 ± 13.4	115.2 ± 117.4	121.0 ± 11.2	117.8 ± 10.3	116.2 ± 13.6	0.641
DBP ([mmHg])	81.1 ± 8.7	79.3 ± 11.3	79.7 ± 5.7	76.5 ± 3.5	74.1 ± 10.2	79.6 ± 67.0	81.2 ± 2.5	76.2 ± 6.8	0.260
PTT [msec]	339.2 ± 24.3	336.3 ± 21.8	336.3 ± 41.1	342.4 ± 24.5	341.4 ± 22.7	341.0 ± 21.0	337.8 ± 16.6	339.2 ± 20.0	0.768
RR [msec]	985.4 ± 164.4	1088.3 ± 189.7	914.1 ± 104.0	1128.7 ± 180.3	1100.9 ± 134.1	1153.3 ± 161.4	1105.8 ± 169.4	981.8 ± 181.8	**0.004**

n. u., normalized units; PR, pulse rate; bpm, beats per minute; HRV-LF/HF, low-to-high frequency spectral power ratio of heart rate variability; SBP, systolic blood pressure; DBP, diastolic blood; mmHg, millimeters of mercury; PTT, pulse transit time; RR, time elapsed between two successive R-waves; msec, milliseconds. Mean values +/− standard deviations are shown. *p*-values for significant differences are plotted in bold.

Notably, PR and HRV LF/HF were significantly elevated during the night of complete sleep deprivation compared to the remaining nights (*p* = 0.001). In addition, descriptive analyses reported slightly higher values during the pre-isolation night (Day −5) and the post-isolation night (Day 120 + 5), indicating a broader pattern of altered autonomic regulation outside the stable isolation phase (Table 3).

### Differences between sleep fragmentation conditions

To further characterize the effects of sleep disruption, the two sleep fragmentation nights were directly compared. This analysis revealed significantly reduced TIB (*p* = 0.031) and TST (*p* = 0.021) during Fragmentation 2 compared to Fragmentation 1.

Despite these differences in sleep duration, no significant differences were observed in ANS state parameters during sleep between the two fragmentation conditions, suggesting comparable autonomic profiles despite varying degrees of sleep loss.

## Discussion

During the four-month isolation experiment of a SAM, parameters of sleep structure and ANS state were recorded across eight individual nights under different sleep conditions. To assess ANS state parameters, a self-applicable, medical-grade sleep recorder was used, which proved suitable for extreme conditions comparable to those of a space mission. In addition to sleep, the recorder enabled continuous measurement of various ANS state parameters. The analyses revealed significant differences between experimental nights for selected parameters. While the ANS of participants under space-like conditions appeared generally resilient to moderate sleep impairment, a night of complete sleep deprivation was associated with increased autonomic activation. Furthermore, the impact on ANS state differed depending on the type of sleep disturbance. Objective assessment of such an autonomic imbalance is important, as it reflects altered autonomic regulation under demanding conditions.

The present findings extend existing literature by demonstrating that, within a prolonged isolation setting, nocturnal ANS regulation appears markedly affected by complete sleep deprivation. Indeed, previous SAM studies investigated HRV as a marker of autonomic activity but did not examine either total sleep loss or short-term disruption in isolation (Vigo et al., 2012; Benítez-Salgado et al., 2024). Importantly, the observed changes in autonomic parameters, including the increased pulse rate and elevated HRV LF/HF ratio during sleep deprivation, indicate a shift toward sympathetic predominance, suggesting not only statistical but also physiologically meaningful alterations in nocturnal autonomic regulation. By directly comparing different types of sleep disturbance within the same protocol, the current study provides novel evidence that the magnitude and nature of autonomic responses depend on the specific form of sleep disruption, highlighting the particular vulnerability of ANS regulation to sustained wakefulness under operational conditions.

### HRV as an indicator of ANS state

HRV is commonly used as an indicator of ANS regulation, as alterations in sympathetic and parasympathetic activity are reflected in changes in heart rate dynamics (Duong et al., 2020; Pizzo et al., 2022). In general, higher HRV reflects greater parasympathetic modulation and autonomic flexibility, which are commonly interpreted as indicators of effective recovery and adaptive regulation. In contrast, reduced HRV or a shift toward sympathetic predominance is associated with sustained physiological activation and diminished recovery capacity (Goel and Dinges, 2012; Tobaldini et al., 2013; Pizzo et al., 2022). Within this framework, the present findings suggest that a night of complete sleep deprivation was associated with a shift toward sympathetic predominance and reduced parasympathetic modulation during the nocturnal period in six healthy participants.

The majority of existing research on ANS responses to sleep deprivation has demonstrated such effects under tightly controlled laboratory conditions (Glos et al., 2014; van Leeuwen et al., 2018; Yang et al., 2019; Schlagintweit et al., 2023). Although these studies have provided valuable mechanistic insights, they are limited in their ability to capture operational and environmental constraints such as prolonged isolation and confinement. SAMs are designed to approximate several aspects of real spaceflight and thereby enhance ecological validity, although certain key environmental factors, most notably microgravity, cannot be fully replicated. As microgravity is known to influence ANS function, for example, by reducing sympathetic drive to maintain cardiac output and blood perfusion stable (Migeotte et al., 2003), SAMs should be viewed as a complementary framework for investigating sleep–ANS interactions under space-relevant conditions (Rai et al., 2012; Demin et al., 2013; Rahman et al., 2022).

Building on these findings, the present study extends existing research by examining autonomic responses not only to complete sleep deprivation but also to experimentally induced sleep fragmentation under prolonged isolation. Although the autonomic consequences of total sleep loss are relatively well established, the effects of shorter, operationally relevant sleep disruptions on nocturnal ANS regulation remain less well understood in space-analogue settings. Assessing nocturnal autonomic regulation under these conditions may therefore provide additional insights into physiological adaptation to different forms of sleep disruption. Interpretation of these findings should take into account that HRV measures are influenced by behavior, respiration, body position, and methodological factors (Penzel et al., 2016).

### Sleep structure and subjective sleepiness

Sleep structure differed between nights, primarily with respect to total sleep time and sleep efficiency. Undisturbed nights during isolation were characterized by longer sleep duration, whereas both sleep fragmentation conditions resulted in reduced sleep efficiency and increased wakefulness. Despite these changes in sleep continuity, sleep stage distribution did not differ significantly across nights.

Subjective sleepiness, assessed using the KSS, did not show significant differences between evening and morning ratings for most study nights. Only following the night without sleep was a trend toward increased morning sleepiness observed, with higher morning than evening KSS scores in four of the six participants, consistent with the individual differences illustrated in Figure 1. This trend did not reach statistical significance and was not observed after nights with sleep fragmentation, suggesting that moderate sleep disruption did not consistently translate into increased subjective sleepiness the following morning.

### ANS state parameters across mission phases

An additional observation from Table 3 is that ANS state parameters during the pre-isolation (Day −5) and post-isolation (Day 120 + 5) nights tended to be less favorable than during undisturbed nights within the isolation phase, as reflected by higher pulse rates and elevated HRV LF/HF ratios. Notably, this pattern occurred despite the absence of experimental sleep disturbance during these nights, suggesting that autonomic regulation was influenced not only by the sleep condition but also by the mission phase and associated contextual factors. In the pre-isolation phase, crew members were exposed to intensive instruction schedules and anticipatory demands related to mission start, which may have contributed to increased autonomic activation and higher self-rated sleepiness levels. Supporting this interpretation, parallel investigations conducted within the interdisciplinary SIRIUS-19 experiment reported an initial increase in hair cortisol levels during the early mission phases, indicative of an adaptation response, while self-rated affective states remained largely positive and stable throughout the mission (Pauly et al., 2025). Similarly, the post-isolation phase may reflect re-adaptation processes following prolonged confinement, which could transiently challenge autonomic stability. In contrast, the isolation phase itself was characterized by a highly structured and predictable daily routine, potentially facilitating physiological adaptation and stabilization of ANS regulation. Comparable phase-dependent patterns have been described in other long-duration space-analogue studies, including the Mars-520 experiment, where behavioral and physiological responses varied across mission phases and transitional periods rather than progressing linearly with time in confinement (Basner et al., 2014).

### Sleep fragmentation versus complete sleep deprivation

Although sleep fragmentation clearly impaired sleep continuity, its effects on average nocturnal ANS state parameters were limited. No significant differences in ANS measures were observed between the two fragmentation conditions, despite differences in total sleep time and time in bed. In contrast, the night of complete sleep deprivation was associated with significantly increased pulse rates and HRV LF/HF ratios, indicating reduced parasympathetic modulation and incomplete nocturnal autonomic recovery. These findings suggest that nocturnal autonomic regulation in this cohort was more sensitive to sustained wakefulness than to isolated episodes of sleep interruption. However, given the small sample size and the assessment of only single nights per condition, subtle effects of sleep fragmentation or cumulative effects across repeated disturbed nights cannot be excluded.

### Complementary to previous space analogue studies of sleep

Previous SAMs have reported alterations in autonomic regulation during prolonged isolation. For example, long-duration confinement experiments, such as the Mars-500 program, including both the 105-day pilot and the 520-day mission, demonstrated shifts in autonomic balance, with evidence of increased parasympathetic activity and altered sleep–wake regulation across confinement periods (Vigo et al., 2012; Basner et al., 2014). More recent short-duration analogue missions have likewise shown dynamic changes in linear and non-linear HRV indices across mission days, suggesting progressive autonomic adaptation to environmental stressors (Benítez-Salgado et al., 2024).

These investigations have provided important insights into autonomic adaptation during isolation, primarily based on wakefulness assessments, discrete measurement periods, or short-term recordings under operational conditions. In contrast, the present investigation focused specifically on nocturnal autonomic regulation during sleep using continuous recordings, thereby capturing ANS dynamics during a physiological state central to recovery and restoration.

Differences in study duration, experimental design, sleep manipulation protocols, measurement techniques, and timing of assessments necessarily limit direct, one-to-one comparability between studies. Rather than aiming to replicate earlier findings, the present results extend existing space analogue research by providing complementary insights into how sleep disruption and sustained wakefulness are reflected in nocturnal autonomic regulation during confinement. Together, these findings contribute to a broader, methodologically diverse understanding of autonomic adaptation in space analogue environments.

### Feasibility of portable sleep and ANS monitoring

A further aim of this study was to evaluate the feasibility of using a portable, self-applicable, medical-grade device for assessing sleep and ANS state under prolonged isolation. Reliable recordings were obtained across all experimental conditions, including nights of sleep deprivation and sleep fragmentation. This demonstrates the technical robustness and practical applicability of such systems in confined and operationally demanding environments, although formal validation against laboratory-based reference methods was beyond the scope of this study.

### Limitations

Several limitations must be considered. The small sample size of six participants limits statistical power and generalizability. In addition, the crew represented a selected volunteer sample, including individuals with prior experience in space-analogue or isolation experiments. This, together with the above-average physical fitness of participants due to mission selection criteria, may have increased familiarity with confinement, operational stress, sleep disruption, and the self-application of recording devices, as well as influenced autonomic regulation (e.g., higher baseline vagal activity) and resilience to sleep disruption. As such, selection bias cannot be excluded, and these factors may have attenuated the observed responses and limit generalizability beyond similarly healthy, motivated, and highly trained individuals. Sleep disturbance conditions were assessed on single nights only, and randomization of conditions was not feasible due to the fixed mission timeline. Period effects and adaptation processes cannot be fully excluded. In addition, daytime performance outcomes were not assessed, limiting conclusions regarding functional consequences. Finally, HRV indices, particularly frequency-domain measures, should be interpreted cautiously under conditions of fragmented sleep and altered respiration, as changes in breathing patterns may influence spectral components independently of autonomic nervous system activity.

## Conclusion

In this exploratory space-analogue study, sleep structure and nocturnal ANS regulation remained largely stable during undisturbed sleep under prolonged isolation. Sleep fragmentation primarily affected sleep continuity, whereas complete sleep deprivation was associated with more pronounced alterations in nocturnal autonomic regulation. These findings suggest that, in healthy and well-trained individuals, nocturnal ANS regulation may be relatively resilient to isolated sleep interruptions but more sensitive to sustained sleep loss. In addition, based on our findings of impaired ANS state during the pre-isolation phase, further exploration and attention to this period may be important, since optimal functioning is required from the first day of space missions. Further studies investigating sleep structure and ANS state with larger samples, repeated disturbance nights, and assessments during real space missions or SAMs are required to substantiate these observations.

## Data Availability

The original contributions presented in the study are included in the article/supplementary material, further inquiries can be directed to the corresponding author.
